# Development and psychometric properties of a new social support scale for self-care in middle-aged patients with type II diabetes (S4-MAD)

**DOI:** 10.1186/1471-2458-12-1035

**Published:** 2012-11-28

**Authors:** Shohreh Naderimagham, Shamsaddin Niknami, Farid Abolhassani, Ebrahim Hajizadeh, Ali Montazeri

**Affiliations:** 1 Department of Health Education, Faculty of Medical Sciences, Tarbiat Modares University, Tehran, Iran; 2 Department of Health Management, National Institute of Health Research, Tehran University of Medical Sciences, Tehran, Iran; 3 Department of Biostatistics, Faculty of Medical Sciences, Tarbiat Modares University, Tehran, Iran; 4 Mental Health Research Group, Health Metrics Research Centre, Iranian Institute for Health Sciences Research, ACECR, Tehran, Iran

## Abstract

**Background:**

Social support has proved to be one of the most effective factors on the success of diabetic self-care. This study aimed to develop a scale for evaluating social support for self-care in middle-aged patients (30–60 years old) with type II diabetes.

**Methods:**

This was a two-phase qualitative and quantitative study. The study was conducted during 2009 to 2011 in Tehran, Iran. In the qualitative part, a sample of diabetic patients participated in four focus group discussions in order to develop a preliminary item pool. Consequently, content and face validity were performed to provide a pre-final version of the questionnaire. Then, in a quantitative study, reliability (internal consistency and test-retest analysis), validity and factor analysis (both exploratory and confirmatory) were performed to assess psychometric properties of the scale.

**Results:**

A 38-item questionnaire was developed through the qualitative phase. It was reduced to a 33-item after content validity. Exploratory factor analysis loaded a 30-item with a five-factor solution (nutrition, physical activity, self monitoring of blood glucose, foot care and smoking) that jointly accounted for 72.3% of observed variance. The confirmatory factor analysis indicated a good fit to the data. The Cronbach’s alpha coefficient showed excellent internal consistency (alpha=0.94), and test-retest of the scale with 2-weeks intervals indicated an appropriate stability for the scale (ICC=0.87).

**Conclusion:**

The findings showed that the designed questionnaire was a valid and reliable instrument for measuring social support for self-care in middle-aged patients with type II diabetes. It is an easy to use questionnaire and contains the most significant diabetes related behaviors that need continuous support for self-care.

## Background

Diabetes is a worldwide health problem and the number of people with diabetes will exceed to 366 million in 2030 and mostly among middle-aged populations [1]. This widespread of the disease makes any health care systems unable to respond to the need of patients [2]. Thus, it is suggested that self-care in this context is essential [3]. Self-care is defined as ‘self-motivation, understanding and considering the situations that influence health, making decisions to improve health, and implementing these decisions’ [4]. However, there is no promise for self-care if we do not accurately recognize and evaluate the constructs that are affective on it. It has been suggested self-care needs some additive components such as social support to be maintained. It has been recommended that providing social support for self-care might have twofold advantages. Firstly, it could prevent the possible complications and secondly, could guarantee the continuity of the self-care behavior where self-management plays an important role in patients’ overall health status [5,6]. Several self-care behaviors were recognized to improve health in diabetic patients. For instance, Mc Dowell et al. believe nutrition, physical activity, self-monitoring of blood glucose (SMBG), and foot care as the main behaviors for self-care in patients with type II diabetes [7]. It is argued that these behaviors could not be achieved unless we provide appropriate social support for patients [8,9]. However, as there are many types of social support [10,11], patients with diabetes also could receive different types of support for self-care such as informational, emotional and instrumental [12-14].

Several studies examined the relationship between self-care and social support in patients with diabetes [9,15,16]. The consistent findings from the literature were that patients with diabetes did not receive enough social support from families and professionals and when they received such supports, they showed improved self-care behaviors [17,18]. Yet, the main critic with previous studies relies on the fact that similar measures were used to assess social support for patients with different demographic backgrounds while, for instance, providing social support for young diabetic patients could be different from providing social support for elderly patients. Even, in a study, it was found that older adolescents have obtained less family support than younger ones, and older girls reported the highest level of support from their friends but the highest level of family support was for younger boys [19].

Therefore, the preliminary aim of this study was to develop a tailored measure for self-care social support. Since most diabetic patients are in the middle years of their lives, we thought to tailor an instrument for this age group. In brief, tailoring is a very useful psychological and social approach that could enhance health behaviors in target population [20]. Hawkins et al. refer tailoring to a number of methods for creating individualized communications that will lead to larger intended effects of these communications [21]. In addition, it was found that existing measures for self-care social support had no focus on all behaviors or included only a specific type of social support. Accordingly, the second aim of this study was to develop a comprehensive instrument that includes all forms of support for at least five recommended self-care behaviors that needs social support.

## Methods

### Scale development

This was a study to develop an instrument to measure social support for self-care in diabetic patients. Evidence suggests that social support makes people in general and patients in particular more able to care about themselves and maintain healthy behaviors. Social support can come from a variety of sources and is defined as the help one receives from family, friends, and significant others (such as physicians) [12,22].

Several procedures were followed to provide an item pool for the study:

i. A review of the literature.

ii. A small-scale qualitative study was conducted to explore what does ‘social support for self-care’ mean to diabetic patients. For the purpose of qualitative phase, four focus group discussions were conducted with a sample of diabetic patients. Patients were recruited from diabetes screening centers affiliated to Tehran University of Medical Sciences. We have tried to recruit patients with different characteristics to ensure that patients from diverse demographic backgrounds are present in the focus groups. In all, 38 patients agreed to take part in the study. The characteristics of patients are presented in Table 1. All patients informed about the aim of the study and their informed consent was obtained. The discussions were hold in the screening center and all were tape-recorded. We stopped data collection until saturation was reached. Then, we transcribed group discussions and used a deductive method to analyze the data. Deductive content analysis is used when the structure of analysis is operationalized on the basis of previous knowledge [23]. Since we were concerned about five main self-care behaviors, the intention was to determine the frequency of sayings under five topics that were nutrition, physical activity, self-monitoring of blood glucose (SMBG), foot care and smoking. Finally, a list of items was prepared with their examples. Trustworthiness of the results also was checked. As suggested four criteria were considered for the trustworthiness: credibility, transferability, dependability and confirmability [24]. For credibility, we designed clear processes for drawing conclusions from the data. For transferability, we provided rich enough descriptions and data sets that other researchers can use them in the other contexts and settings. For dependability, we checked the consistency of the study processes and finally, we checked the internal coherence of the data and the findings for confirmability [24].

iii. Interview with a panel of experts: Experts were asked ‘What are the most important self-care behaviors in type 2 diabetics? Why these behaviors are so important? Why do you think the other behaviors are not as much important as your selected behaviors?’

**Table 1:** Demographic profile of the study participants

	**Qualitative sample (n = 38)**		**Sample for EFA (n = 204)**		**Sample for CFA (n = 138)**	
	**Mean (SD)**	**No. (%)**	**Mean (SD)**	**No. (%)**	**Mean (SD)**	**No. (%)**
**Age**	45.7 (7.3)		50. 6 (7.5)		50.8 (7.2)	
**Gender**						
Female		25 (65.8)		149 (73)		107 (77.5)
Male		13 (34.2)		55 (27)		31 (22.5)
**Education**						
Illiterate		10 (26.3)		38 (18.6)		30 (21.7)
Primary & secondary		21 (55.3)		140 (68.6)		88 (63.8)
High school graduated		6 (15.8)		23 (11.3)		15 (10.9)
Higher		1 (2.6)		3 (1.5)		5 (3.6)
**Marital Status**						
Single		2 (5.3)		4 (2)		2 (1.5)
Married		34 (89.4)		175 (85.8)		119 (86.2)
Divorced or Widow		2 (5.3)		25 (12.2)		17 (12.3)
**Employment**						
Housewife		18 (47.4)		132 (64.7)		98 (71)
Employed		8 (21)		49 (24)		36 (26.1)
Student		2 (5.4)		0 (0)		0 (0)
Unemployed		5 (13.1)		3 (1.5)		1 (0.7)
Retired		5 (13.1)		20 (9.8)		3 (2.2)
**BMI (Kg/m**^**2**^**)**	29.6 (4.2)		29.7 (4.2)		30 (4.8)	
**Disease duration (year)**	8.2 (5.4)		9.1 (5.6)		11.1 (5.7)	

At last, the data derived from the literature, the qualitative phase and interviews were crosschecked and in all 38 items were generated. Consequently content and face validity were evaluated.

*Content validity:* is a comprehensive review by an expert panel to decide whether items adequately cover the behavior that you are interested in measuring them [25]. It is an essential step for developing a scale and a mechanism for linking abstract concepts with tangible and measurable indicators [26]. The expert panel was consisted of 12 specialists in health education, nursing and internal medicine. Qualitative content validity was determined based on ‘grammar’, ‘wording’, ‘item allocation’, and ‘scaling’ indices [27]. All items were checked and the expert panel’s recommendations were inserted into the questionnaire. Content Validity Ratio (CVR) and Content Validity Index (CVI) were calculated in order to perform quantitative content validity. For calculating CVR, the expert panel was asked to evaluate each item using a 3-point Likert scale: 1 = essential, 2 = useful but not essential and 3 = unessential. Then, according to Lawshe’s table [28], items with CVR score of 0.56 or above were selected [27]. For the CVI, based on Waltz and Bausell [29] recommendation, the same panel was asked to evaluate the items according to a 4-point Likert scale on ‘relevancy’, ‘clarity’, and ‘simplicity’. A CVI score of 0.80 or above was considered satisfactory [30].

*Face validity:* is an evaluation of lay people in understanding and comprehending a scale [25]. In this part, both quantitative and qualitative methods were applied. For quantitative part, 10 patients were asked to evaluate the questionnaire and score the importance of each item on a 5-point Liker scale in order to calculate ‘Item Impact Score’ (Impact Score = Frequency (%) × Importance). The impact score of 1.5 or above was considered satisfactory as recommended [27]. For the qualitative part, the same patients were asked about the ‘relevancy’, ‘ambiguity’, and ‘difficulty’ of the items; and some minor changes were made to the preliminary questionnaire.

*Pre-final version:* following the reflection of the above approaches, finally 5 items were removed and the pre-final version of the questionnaire consisting of 33 items was provided for the next stages (validity and reliability of the questionnaire).

### The main study and data collection

A cross sectional study was designed to evaluate the psychometric properties of the S4-MAD. A consecutive sample of the middle-aged patients with type II diabetes was recruited from two screening diabetes centers affiliated to Tehran University of Medical Sciences. The patients were entered into the study if they were aged between 30 to 60 years, their last HbA1C test was equal or above 7% (or their FBS test was more than 150 mg/dl), having diabetes for more than 1 year, and wished to participate in the study.

### Statistical analysis

#### Validity

The construct validity of the questionnaire was performed using both exploratory (EFA) and confirmatory factor analyses (CFA).

a) *Exploratory factor analysis (EFA):* A sample of 204 patients completed the questionnaire and its factor structure was extracted using the principal component analysis with varimax rotation. The Kaiser-Meyer-Olkin (KMO) and Bartlett’s Test of Sphericity were used to assess the appropriateness of the sample for the factor analysis. Eigenvalues above 1 and scree plot were used for determining the number of factors. Factor loadings equal or greater than 0.4 were considered appropriate [31].

b) *Confirmatory factor analysis (CFA):* A separate sample of 138 patients completed the questionnaire and factor analysis was performed for assessing the model fitness. As recommended various fit indices including relative Chi-square (*χ*^2^/df), Comparative Fit Index (CFI), Incremental Fit Index (IFI), Normed Fit Index (NFI), Non-Normed Fit Index (NNFI), Root Mean Square Error of Approximation (RMSEA), and Standardized Root Mean Square Residual (SRMR) were used [32]. Relative Chi-square is the ratio of chi-square to degrees of freedom and its recommended reference value is less than 3 for accepting the fitness of the model [33]. The value for CFI, IFI, NFI and NNFI could range between ‘0 to 1’ and values closer to 1 are indicative of data fitness [34]. An RMSEA ranged 0.08 to 0.10 shows a mediocre fit and below 0.08 indicates a good fit [35]. The acceptable value for SRMR is less than 0.10 where values less than 0.08 indicate adequate fit and values below 0.05 indicate good fit [36,37].

### Reliability

Internal consistency was evaluated by Cronbach’s α coefficient. Cronbach’s α coefficient of 0.7 or above was considered satisfactory [38,39]. In addition, a sub-sample of patients (n = 15) completed the questionnaire twice with a 2-weeks interval in order to examine the stability of the scale by calculating Intraclass Correlation Coefficient (ICC) where the ICC of 0.4 or above was considered acceptable [40].

All statistical analyses except confirmatory factor analysis were performed using the SPSS version 16.0 [41]. The confirmatory factory analysis was performed using the LISREL 8.80 for Windows [42].

### Ethics

The ethics committee of Tarbiat Modares University approved the study. All patients gave their written informed consent.

## Results

### Participants

In all, 342 diabetic patients participated in this study. Of these, 204 patients took part in the main study and the remaining 138 patients completed the questionnaire in order to perform confirmatory factor analysis. The characteristics of the patients are shown in the Table 1.

### Exploratory factor analysis

The Kaiser-Meyer-Olkin was 0.92, and the Bartlett’s test of sphericity was significant (5.55, P<0.001) showing sampling adequacy. The initial analysis indicated a 5-factor structure for the questionnaire with 3 items loading unexpectedly and irrelevant to the loaded construct. Thus, these were removed from the analysis and a final 30-item questionnaire loaded on five distinct constructs that jointly accounted for 72.3% of variance observed (Table 2) [Additional files 1 and 2].

**Table 2:** The social-support scale for self-care in middle-aged patients with type 2 diabetes and its factors loading (n=204)

**I HAVE….**					
**Item**	**Factor 1**	**Factor 2**	**Factor 3**	**Factor 4**	**Factor 5**
1. Somebody who encourages me to keep the recommended diet by my physician or nutritionist.	0.181	**0.737**	0.059	0.215	−0.077
2. Somebody who shows how happy she/he is when I keep the recommended diet by my physician or nutritionist.	0.200	**0.807**	0.165	0.057	0.034
3. Somebody who buys the necessary ingredients to cook appropriate foods for diabetics.	0.317	**0.615**	0.234	0.095	0.098
4. Somebody who helps me to schedule for eating meals and snacks.	0.432	**0.486**	0.426	0.160	0.069
5. Somebody who cooks appropriate foods for a diabetic patient for me.	0.331	**0.728**	0.192	0.190	0.041
6. Somebody who warns me when I eat more or less than of my eating plan.	0.280	**0.753**	0.212	0.287	0.012
7. Somebody who eats the foods that I can eat so that I do not have any temptation and can go on my diet.	0.141	**0.681**	0.125	0.155	−0.083
8. Somebody who –before any meal or snack- tells me the ingredients of that food are appropriate for me or not.	0.010	**0.524**	0.230	0.114	0.026
9. Somebody who reminds me repeatedly about the necessity of continuing my diet.	0.156	**0.673**	0.404	0.153	0.105
10. Somebody who encourages me to have physical activity regularly.	0.348	0.338	0.279	**0.590**	0.036
11. Somebody who reminds me about various methods of physical activity (exercise, job or household activities).	0.261	0.409	0.237	**0.637**	0.051
12. Somebody who pays the cost of registering in a gym or buying equipment for physical activity.	0.249	0.154	0.096	**0.835**	−0.039
13. Somebody who reminds me that I must have more physical activity when I am lazy.	0.233	0.331	0.120	**0.747**	−0.063
14. Somebody who asks me to join him/her for exercise.	−0.016	0.094	0.151	**0.837**	0.104
15. Somebody who always asks me about the result of my blood glucose test.	0.059	0.434	**0.577**	0.247	0.110
16. Somebody who pays attention and reads the amount of my blood glucose from the glucometer while self-monitoring of blood glucose.	0.217	0.197	**0.834**	0.164	−0.030
17. Somebody who helps me to monitor the glucose of my blood by glucometer when I am not strong enough.	0.307	0.195	**0.794**	0.128	−0.010
18. Somebody who reminds me about the time of blood glucose test in laboratory every 3 months.	0.503	0.320	**0.605**	0.172	0.018
19. Somebody who checks all the necessary equipment to perform Self-Monitoring of Blood Glucose.	0.360	0.267	**0.744**	0.126	0.008
20. Somebody who encourages me to perform Self-Monitoring of Blood Glucose independently.	0.427	0.309	**0.553**	0.117	0.074
21. Somebody who pays attention to the signs of hypoglycemia in me.	0.476	0.251	**0.651**	0.153	0.049
22. Somebody who gives me educational materials (CD, book and etc.) about foot care in diabetics.	**0.718**	0.149	0.307	0.231	0.164
23. Somebody who reminds me of the daily foot care.	**0.802**	0.201	0.242	0.113	0.192
24. Somebody who encourages me to perform daily foot care.	**0.815**	0.278	0.244	0.101	0.108
25. Somebody who performs daily foot care for me when I am not strong enough.	**0.796**	0.130	0.308	0.181	0.058
26. Somebody who always makes sure that all necessary things for foot care such as warm water and mild soap are available.	**0.728**	0.335	0.130	0.230	0.064
27. Somebody who helps me with foot care.	**0.784**	0.232	0.253	0.123	0.195
28. Somebody who helps and encourages me to quit smoking.	0.135	0.006	0.030	0.048	**0.954**
29. Somebody who registers me in a smoke quitting center.	0.148	0.002	0.025	0.003	**0.970**
30. Somebody who gives me educational materials (CD, book and etc.) about smoking and its effects on diabetics.	0.171	0.027	0.017	0.006	**0.948**
**Eigenvalue**	*13.4*	*3.1*	*2.1*	*1.7*	*1.4*
**Explained Variance (%)**	*44.7*	*10.4*	*6.8*	*5.8*	*4.6*
**Cumulative Variance (%)**	*44.7*	*55.1*	*61.9*	*67.7*	*72.3*

Factor 1: Foot care, Factor 2: Nutrition, Factor 3: Self-monitoring of blood glucose, Factor 4: Physical activity, Factor 5: Smoking.

### Confirmatory factor analysis

**Figure 1:**
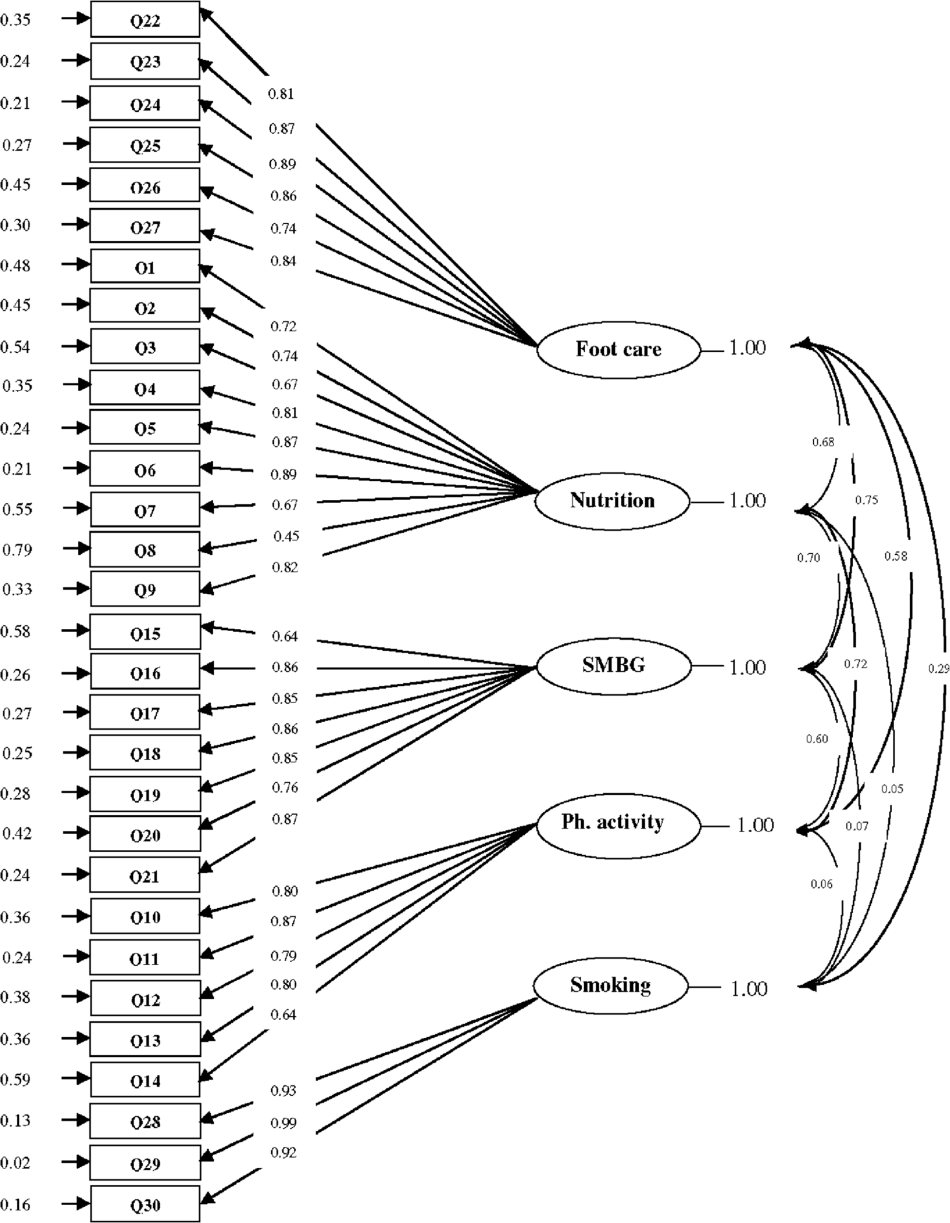
A five-factor model for the questionnaire obtained from confirmatory factory analysis (n = 138).

The 30-item questionnaire was subjected to the confirmatory factor analysis. The relative chi-square (*χ*^2^/*df*) was 2.03 indicating the fitness of the model (P<0.0001). All comparative indices of the model including CFI, IFI, NFI and NNFI were more than 0.9 (0.96, 0.96, 0.93 and 0.96 respectively) showing the goodness of fit for the data. The RMSEA of the model was 0.087 (90% CI=0.078-0.096). The SRMR was less than 0.08 (0.06) confirming an adequate fit for the model. The results obtained from the CFA are presented in Figure 1.

### Reliability

The instrument had an excellent internal consistency (0.94). The intraclass correlation coefficient was 0.87 indicating an appropriate stability of the questionnaire. The results are shown in Table 3.

**Table 3:** Cronbach’s α coefficient and ICC for the social support scale and its subscales (n =204)

	**Number of items**	**Mean (SD)**	**Cronbach’s α coefficient**	**ICC (n= 15)**
Nutrition	9	2.86 (1.54)	0.89	0.87
Physical activity	5	2.45 (1.44)	0.88	0.83
SMBG	7	2.64 (1.60)	0.92	0.89
Foot care	6	2.17 (1.47)	0.94	0.93
Smoking	3	0.75 (1.36)	0.97	0.91
**Total**	**30**	**2.39 (1.50)**	**0.94**	**0.87**

## Discussion

The present study reported the stages of designing and developing a scale for evaluating social support for self-care in middle-aged patients with type II diabetes and the findings indicated satisfactory psychometric properties for the questionnaire. Social support for self-care is a crucial issue in adults with diabetes [18]. It can contribute to treatment adherence and the outcomes. For instance, evidence suggests that encouraging families to exercise with their diabetic patients could increase physical activity among these people [17].

There are a number of scales for measuring social support [43-46] but to the authors’ best knowledge, there are only two questionnaires for assessing social support in diabetic patients the Diabetes Social Support Questionnaire, and the Diabetes Care Profile (DCP) [47-49]. The DSSQ is a well-known instrument and has been used in a number of studies on social support for self-care in diabetic patients [47,50]. It was primarily developed for type I diabetic patients and measures insulin injection, blood glucose testing, meal plan, exercise and emotional support. The Diabetes Care Profile (DCP) is a set of different sections and one section including six questions related to social support. The DCP asks the support that one receives for meal plan, medicine, foot care, physical activity, testing blood sugar and emotional item [48]. However, the focus of current study was to develop a scale containing the five most important diabetes related behaviors namely nutrition, physical activity, self-monitoring of blood glucose (SMBG), foot care and smoking. It is argued that when addressing self-care activities in diabetic patients, it is important to address the unmet needs for social support [49]. As different aspects of social support for self-care in diabetes patients might be considered, it is recommended that when assessing social support, there is need to decide which aspects of support are relevant to measure in any specific situation [51]. In this study, we thought diabetic patients need instrumental, informational and emotional support for self-care and thus, we considered these three aspects of social support and incorporated them to the all dimensions of the questionnaire as needed.

Performing both exploratory and factor analyses, the results indicated a good structure for this new instrument. Exploratory factor analysis indicated that the five-factor structure of the questionnaire could jointly account for 72.3% of the total observed variance. It seems that a careful selection of items related to social support for self-care might be the reason why we obtained such satisfactory results [52]. CFA also showed that factor structure of this scale was appropriate.

A reliable instrument can increase the power of the study to recognize real significant correlations and differences in the study [53]. Internal consistency of the final scale as measured by the Cronbach’s alpha coefficient was found to be 0.94 indicating a desirable reliability. In addition, ICC showed appropriate stability for the scale as it was examined by 15 patients with a 2-weeks interval (0.87).

The main feature of the S4-MAD was the fact that it was developed for middle age diabetic patients (the main affected age group), and contained items on foot care and smoking. However, we did not include items on medications since neither patients nor experts did address the topic during the course of scale development. In addition a unique item (item 18) was included in this new instrument on reminding patients for blood glucose test every three months. The other characteristic of our questionnaire was related to its wording structure. Unlike other questionnaires that contain short statements for each item we used prolonged and complete sentences in order to help patients to understand the items and avoid confusion. Finally, we believe without losing any important dimension on social support for self-care, the S4-MAD is relatively a short questionnaire and easy to use.

This study however had few limitations. For example we did not perform concurrent validity in order to demonstrate the instrument correlates well with a measure that has previously been validated in Iran. Yet, the study had a number of strengths. Notably we recruited two separate samples for the study. In fact, as recommended, we used one sample for the EFA and another sample for the CFA.

## Conclusion

The social support scale for self-care in middle-aged patients with type II diabetes is a valid and reliable instrument for evaluating the social support in these patients and now can be used in future studies of social support in patients with type II diabetes.

## Competing interests

The authors declare that they have no competing interests.

## Authors’ contributions

SHNM was the main investigator, collected the data, performed the statistical analysis, and drafted the manuscript. SHN supervised the project and contributed to all aspects of the study. FA, and EH were the study consultants. AM helped as a consultant and contributed to the study design, analysis, provided the final draft, and revised the final version. All authors read and approved the final manuscript.

## Pre-publication history

The pre-publication history for this paper can be accessed here:

http://www.biomedcentral.com/1471-2458/12/1035/prepub

## Supplementary Material

Additional file 1 **The S4-MAD.** The file contains the Social Support Scale for Self-Care in Middle-Aged Patients with Type II Diabetes. Click here for file

Additional file 2 **Scoring Instruction.** The file contains the scoring instruction for the Social Support Scale for Self-Care in Middle-Aged Patients with Type II Diabetes. Click here for file
