# Supplementary material for: Development and psychometric properties of a new social support scale for self-care in middle-aged patients with type II diabetes (S4-MAD)
Source: BMC Public Health. 2012 Nov 28;12:1035. doi: 10.1186/1471-2458-12-1035 (PMC3520699; doi:10.1186/1471-2458-12-1035)
Supplement: Additional file 1 — The S4-MAD. The file contains the Social Support Scale for Self-Care in Middle-Aged Patients with Type II Diabetes. [file 1471-2458-12-1035-S1.doc]

**Social Support Scale for Self-care in Middle-Aged Patients with Type II Diabetes**

**(S4-MAD)**

ID: ………………

Date: …………….

**Instruction**

This questionnaire deals with perceptions that you have about receiving social support from your family, friends and important others (such as physician) for your nutrition, physical activity, self-monitoring of blood glucose, foot care and smoking self-care behaviors. For each question, put a check mark or cross in the box in front of the answer that best describes your beliefs or feelings. Please answer all questions.

| **I HAVE….**  1. Somebody who encourages me to keep the diet recommended by my physician or nutritionist. | |
| --- | --- |
| 1) Never |  |
| 2) Rarely |  |
| 3) Some times |  |
| 4) Often |  |
| 5) Always |  |
| 2. Somebody who shows how happy she/he is when I keep the diet recommended by my physician or nutritionist. | |
| 1) Never |  |
| 2) Rarely |  |
| 3) Some times |  |
| 4) Often |  |
| 5) Always |  |
| 3. Somebody who buys the necessary ingredients to cook appropriate foods for diabetics. | |
| 1) Never |  |
| 2) Rarely |  |
| 3) Some times |  |
| 4) Often |  |
| 5) Always |  |
| 4. Somebody who helps me to schedule for eating meals and snacks. | |
| 1) Never |  |
| 2) Rarely |  |
| 3) Some times |  |
| 4) Often |  |
| 5) Always |  |
| 5. Somebody who cooks appropriate foods for a diabetic patient for me. | |
| 1) Never |  |
| 2) Rarely |  |
| 3) Some times |  |
| 4) Often |  |
| 5) Always |  |
| 6. Somebody who warns me when I eat more or less than of my eating plan. | |
| 1) Never |  |
| 2) Rarely |  |
| 3) Some times |  |
| 4) Often |  |
| 5) Always |  |
| 7. Somebody who eats the foods that I can eat so that I do not have any temptation and can go on my diet. | |
| 1) Never |  |
| 2) Rarely |  |
| 3) Some times |  |
| 4) Often |  |
| 5) Always |  |
| 8. Somebody who –before any meal or snack- tells me the ingredients of that food are appropriate for me or not. | |
| 1) Never |  |
| 2) Rarely |  |
| 3) Some times |  |
| 4) Often |  |
| 5) Always |  |
| 9. Somebody who reminds me repeatedly about the necessity of continuing my diet. | |
| 1) Never |  |
| 2) Rarely |  |
| 3) Some times |  |
| 4) Often |  |
| 5) Always |  |
| 10. Somebody who encourages me to have physical activity regularly. | |
| 1) Never |  |
| 2) Rarely |  |
| 3) Some times |  |
| 4) Often |  |
| 5) Always |  |
| 11. Somebody who reminds me about various methods of physical activity (exercise, job or household activities). | |
| 1) Never |  |
| 2) Rarely |  |
| 3) Some times |  |
| 4) Often |  |
| 5) Always |  |
| 12. Somebody who pays the cost of registering in a gym or buying equipments for physical activity. | |
| 1) Never |  |
| 2) Rarely |  |
| 3) Some times |  |
| 4) Often |  |
| 5) Always |  |
| 13. Somebody who reminds me that I must have more physical activity when I am lazy. | |
| 1) Never |  |
| 2) Rarely |  |
| 3) Some times |  |
| 4) Often |  |
| 5) Always |  |
| 14. Somebody who asks me to join him/her for exercise. | |
| 1) Never |  |
| 2) Rarely |  |
| 3) Some times |  |
| 4) Often |  |
| 5) Always |  |
| 15. Somebody who always asks me about the result of my blood glucose test. | |
| 1) Never |  |
| 2) Rarely |  |
| 3) Some times |  |
| 4) Often |  |
| 5) Always |  |
| 16. Somebody who pays attention and reads the amount of my blood glucose from the glucometer while self-monitoring of blood glucose. | |
| 1) Never |  |
| 2) Rarely |  |
| 3) Some times |  |
| 4) Often |  |
| 5) Always |  |
| 17. Somebody who helps me to monitor the glucose of my blood by glucometer when I’m not strong enough. | |
| 1) Never |  |
| 2) Rarely |  |
| 3) Some times |  |
| 4) Often |  |
| 5) Always |  |
| 18. Somebody who reminds me about the time of blood glucose test in laboratory every 3 months. | |
| 1) Never |  |
| 2) Rarely |  |
| 3) Some times |  |
| 4) Often |  |
| 5) Always |  |
| 19. Somebody who checks all the necessary equipments to perform Self-Monitoring of Blood Glucose. | |
| 1) Never |  |
| 2) Rarely |  |
| 3) Some times |  |
| 4) Often |  |
| 5) Always |  |
| 20. Somebody who encourages me to perform Self-Monitoring of Blood Glucose independently. | |
| 1) Never |  |
| 2) Rarely |  |
| 3) Some times |  |
| 4) Often |  |
| 5) Always |  |
| 21. Somebody who pays attention to the signs of hypoglycemia in me. | |
| 1) Never |  |
| 2) Rarely |  |
| 3) Some times |  |
| 4) Often |  |
| 5) Always |  |
| 22. Somebody who gives me educational materials (CD, book and etc.) about foot care in diabetics. | |
| 1) Never |  |
| 2) Rarely |  |
| 3) Some times |  |
| 4) Often |  |
| 5) Always |  |
| 23. Somebody who reminds me of the daily foot care. | |
| 1) Never |  |
| 2) Rarely |  |
| 3) Some times |  |
| 4) Often |  |
| 5) Always |  |
| 24. Somebody who encourages me to perform daily foot care. | |
| 1) Never |  |
| 2) Rarely |  |
| 3) Some times |  |
| 4) Often |  |
| 5) Always |  |
| 25. Somebody who performs daily foot care for me when I am not strong enough. | |
| 1) Never |  |
| 2) Rarely |  |
| 3) Some times |  |
| 4) Often |  |
| 5) Always |  |
| 26. Somebody who always makes sure that all necessary things for foot care such as warm water and mild soap are available. | |
| 1) Never |  |
| 2) Rarely |  |
| 3) Some times |  |
| 4) Often |  |
| 5) Always |  |
| 27. Somebody who helps me with foot care. | |
| 1) Never |  |
| 2) Rarely |  |
| 3) Some times |  |
| 4) Often |  |
| 5) Always |  |
| 28. Somebody who helps and encourages me to quit smoking. | |
| 1) Never |  |
| 2) Rarely |  |
| 3) Some times |  |
| 4) Often |  |
| 5) Always |  |
| 29. Somebody who registers me in a smoke-quitting-center. | |
| 1) Never |  |
| 2) Rarely |  |
| 3) Some times |  |
| 4) Often |  |
| 5) Always |  |
| 30. Somebody who gives me educational materials (CD, book and etc.) about smoking and its effects on diabetics. | |
| 1) Never |  |
| 2) Rarely |  |
| 3) Some times |  |
| 4) Often |  |
| 5) Always |  |
|  |  |
|  | |
| **Thank you for completing the questionnaire** | |

© Naderi Magham SH, et al., 2012
