# Supplementary material for: Development and psychometric properties of a new social support scale for self-care in middle-aged patients with type II diabetes (S4-MAD)
Source: BMC Public Health. 2012 Nov 28;12:1035. doi: 10.1186/1471-2458-12-1035 (PMC3520699; doi:10.1186/1471-2458-12-1035)
Supplement: Additional file 2 — Scoring Instruction. The file contains the scoring instruction for the Social Support Scale for Self-Care in Middle-Aged Patients with Type II Diabetes. [file 1471-2458-12-1035-S2.doc]

# Scoring Instruction for the Social Support for Self-care in Middle Aged Patients with Type II Diabetes (S4-MAD)

The S4-MAD includes five subscales namely: nutrition (items 1-9), physical activity (items 10-14), self-monitoring of blood glucose (items 15-21), foot care (items 22-27), and smoking (items 28-30).

To calculate the scores please fallow this instruction:

1. Score all items from 1 to 5. These are row scores for each item.

2. To calculate the row score for each subscale, add item raw scores and then divide it to number of items in that subscale.

3. To transfer row scores to a score ranging from 0 to 100, then, use the following formula to calculate the final score:

The subscale score = [(subscale row score–1)/4] × 100

**Example 1:**

To calculate social support for physical activity (item 10 to 14) for someone who scored item 10 as ‘never’, item 11 as ‘always’, item 12 as ‘rarely, item 13 as ‘always’ and item 14 as ‘sometimes’; his/her score for these items would be 1, 5, 2, 5 and 3 respectively. The row score for this subscale then would be:

1+5+2+5+3/5: 3.2

Then, this should be linearly transformed:

[(3.2-1)/4] × 100 = 55

**Example2:**

To calculate social support for foot care for someone who scored item 22 as ‘always’, item 23 as ‘sometimes’, item 24 as ‘rarely’, item 25 as ‘sometimes’, item 26 as ‘always’ and item 27 as ‘never’, his/her score for these items would be 5, 3, 2, 3, 5 and 1 respectively. The row score for this subscale, then, would be:

5+3+2+3+5+1/6: 3.16

Then this should be linearly transformed:

[(3.16-1)/4] × 100 = 54
